# Identifying Hub Genes and miRNA-mRNA Regulatory Networks in Mice Infected with H1N1 Influenza Virus

**DOI:** 10.1155/2023/2291051

**Published:** 2023-05-16

**Authors:** Mingyang Li, Qizhi He, Lingli Chen

**Affiliations:** ^1^Institute of Medical Biology, Chinese Academy of Medical Sciences & Peking Union Medical College, Kunming, Yunnan, China; ^2^School of Basic Medical Science, Changsha Medical University, Changsha, Hunan, China; ^3^Hunan University of Chinese Medicine, Changsha, Hunan, China

## Abstract

H1N1 influenza virus is a major factor in seasonal influenza outbreaks. After the body is infected with the influenza virus, the expression of certain mRNAs, including miRNAs, could be affected. However, the association between these mRNAs and miRNAs remains unclear. This study is aimed at identifying differentially expressed genes (DEGs) and miRNAs (DEmiRs) caused by H1N1 influenza virus infection and constructing a miRNA-mRNA regulatory network. Nine GSE datasets were downloaded from the Gene Expression Omnibus database, of which seven were mRNA data and two were miRNA data. The limma package in R language package was used to analyze array data, and edgeR package was used to analyze high-throughput sequencing data. At the same time, the genes related to H1N1 infection were further screened by WGCNA analysis. DEGs were subjected to Gene Ontology and KEGG pathway enrichment analyses by DAVID database, while the STRING database predicted the protein-protein interaction (PPI) network. The correspondence between miRNA and target mRNA was analyzed by the miRWalk database. Cytoscape software was used to output PPI results, identify hub genes, and construct a miRNA-mRNA regulatory network. 114 DEGs and 37 candidate DEmiRs were identified for subsequent analysis. These DEGs were significantly enriched in response to the virus, cytokine activity, and symbiont-containing vacuole membrane. According to KEGG analysis, DEGs were enriched in PD-L1 expression and PD-1 checkpoint pathway. The key point Cd274 (PD-L1) was highly expressed in the H1N1-infected group. Finally, a potential miRNA-mRNA regulatory network (containing 8 candidate DEmiRs and 69 candidate DEGs) and a PPI network were constructed. After that, three hub genes were identified: Ifit3, Stat2, and Irf7. These hub genes and Cd274 were validated by another independent high-throughput dataset and were highly expressed pattern. This study will help researchers gain insights into the intrinsic effects of H1N1 influenza virus infection on the host and suggest a novel association of H1N1 virus with the host immune system.

## 1. Introduction

The influenza A virus belongs to the Orthomyxoviridae family, and its genome consists of eight single-negative stranded RNAs, encoding a total of 11 viral proteins [1]. According to the antigenic properties of hemagglutinin (HA) and neuraminidase (NA) on the surface of the influenza A virus, it is divided into different subtypes, including 16 HA subtypes and nine NA subtypes [2]. The influenza A virus genome mutation results in a small variation in the antigen called antigenic drift, which is associated with the occurrence of seasonal influenza in humans. The rearrangement of the viral genome leads to a substantial change in the antigen, called antigenic shift, which is often the main cause of influenza pandemics [3]. The seasonal epidemic of the influenza virus causes about 500,000 deaths per year [4], so the prevention of the influenza A virus is highly valued. There is evidence that the serum levels of IL-6, IL-8, IL-15, and TNF*α* were significantly elevated in patients with influenza A virus H1N1 infection, so these cytokines are also used as important diagnostic indicators [5].

The pathogenicity of the influenza A virus is related to viral load and inflammatory factor storm [6]. Intracellular influenza virus RNA initiates the innate immune response to the virus through three intracellular immune pathways: retinoic acid-inducible gene-1 receptor (RIG-1), Toll-like receptor 3, and inflammasome [7]. Research on virus-host interactions, host inflammatory pathways, host innate immunity, and acquired immunity may provide new targets for anti-influenza virus therapy [8].

MicroRNAs (miRNAs), a group of noncoding small RNAs, are important regulators of pathogen-host interactions and play important roles in inflammation and immune responses [9]. Gene products of Epstein-Barr virus latency, such as EBERS, BARF-0, EBNA-1, and LMP2A, directly lead to downregulation of the host cell miR-200 family, critical for EBV-associated gastric cancer development [10].

Although scientists classify influenza A viruses by hemagglutinin and ceramidase, we have always believed that each strain has its own characteristics. An in-depth study of the expression differences of host genes after virus infection and analysis of internal regulatory networks are of great significance for antiviral therapy. For influenza A virus H1N1 infection, the host-intrinsic gene changes, and related miRNA-mRNA regulatory networks remain unclear. Therefore, this study analyzed multiple samples infected by various H1N1 virus strains to screen for identifying DEGs and miRNAs, and constructing a miRNA-mRNA regulatory network will help us understand the intrinsic effects of H1N1 on host cells.

## 2. Materials and Methods

### 2.1. Microarray Data

The mRNA expression profiles GSE31022 [11], GSE36328 [12, 13], GSE54048 [13], GSE40091 [14], GSE69945 [15], and GSE70445 [16] and miRNA expression profiles GSE69944 and GSE62495 were downloaded from the GEO database. The data were extracted from GSE datasets, which contained 139 samples (mouse infected with multiple H1H1 influenza virus strains) and 59 mock infection samples. We normalized these data using the quantile method.

### 2.2. High-Throughput Sequencing Data

GSE98527 was datasets gathered by high-throughput sequencing, involving 15 H1N1-infected mouse samples and three mock samples. These data were normalized by the function log2 (counts + 1).

### 2.3. Identification of DEGs and miRNAs

In order to ensure that batch effects were not a problem, all data were preprocessed [17]. We analyzed microarray data using limma package (version 3.40.6) and high-throughput sequencing data using the edgeR package (H1H1 influenza virus strain-infected group vs. mock-infected group). DEGs and miRNAs were identified with a |Fold Change| ≥ 1. Adjusted *p* value cutoff < 0.05 was defined as statistically significant.

### 2.4. Gene Ontology and Pathway Enrichment Analysis

Gene Ontology (GO) analysis is a widely used method for annotating genes and gene products and identifying characteristic biological attributes for high-throughput genome or transcription data. To analyze the genes at the functional level, GO enrichment and KEGG pathway analyses were performed using DAVID online tool (https://david.ncifcrf.gov/). *p* < 0.05 was considered statistically significant.

### 2.5. Weighted Gene Coexpression Network Analysis

The dataset was analyzed according to the methods published by the contributors to the WGCNA package [18]. The best soft threshold power was set to identify the module-trait relationship, module membership (MM), and gene significance (GS) [19].

### 2.6. Integration of the Protein-Protein Interaction (PPI) Network

To evaluate the interactive relationships between genes, we input the gene list into the STRING (http://www.string-db.org/).

### 2.7. Analysis of Hub Genes

We used CytoHubba (a Cytoscape built-in app) to analyze the hub gene; the top three genes were the resulting output.

### 2.8. miRNA-mRNA Network Construction

miRWalk is a comprehensive miRNA target gene database that compares prediction results with 12 other miRNA databases (DIANAmicroTv4.0, DIANA-microT-CDS, miRanda-rel2010, mirBridge, miRDB4.0, miRmap, miRNAMap, and doRiNA, i.e., PicTar2, PITA RNA22v2, RNAhybrid2.1, and Targetscan6.2). We employed miRWalk (version 3.0) to predict the targets of screened miRNAs, and the output results were intersected with the list of hub genes [20].

### 2.9. Software Environment and Data Visualization

A software installation was downloaded, and R (version 4.2.1) was installed as described at https://www.r-project.org/. Both R and RStudio must be installed. All R analysis and visualization packages are freely accessible from the library.

The miRNA-mRNA network and PPI network data were automatically output by online tools and saved in txt format as nodes and edges. The txt files were exported into Cytoscape software (version 3.9.1), respectively. The visualization results were output after adjusting the layout.

The ggplot2 R package (version 3.3.3) and UpSetR package (Version 1.4.0) were used to show the other analysis results.

## 3. Results

### 3.1. Extracting H1N1 Influenza Virus-Infected Mouse Samples and Moving Batch Effects

We searched for samples from GEO database, and eight microarray datasets were screened. We extracted H1N1 influenza virus-infected mouse samples and mock-infected samples (Table 1).

**Figure 1:**
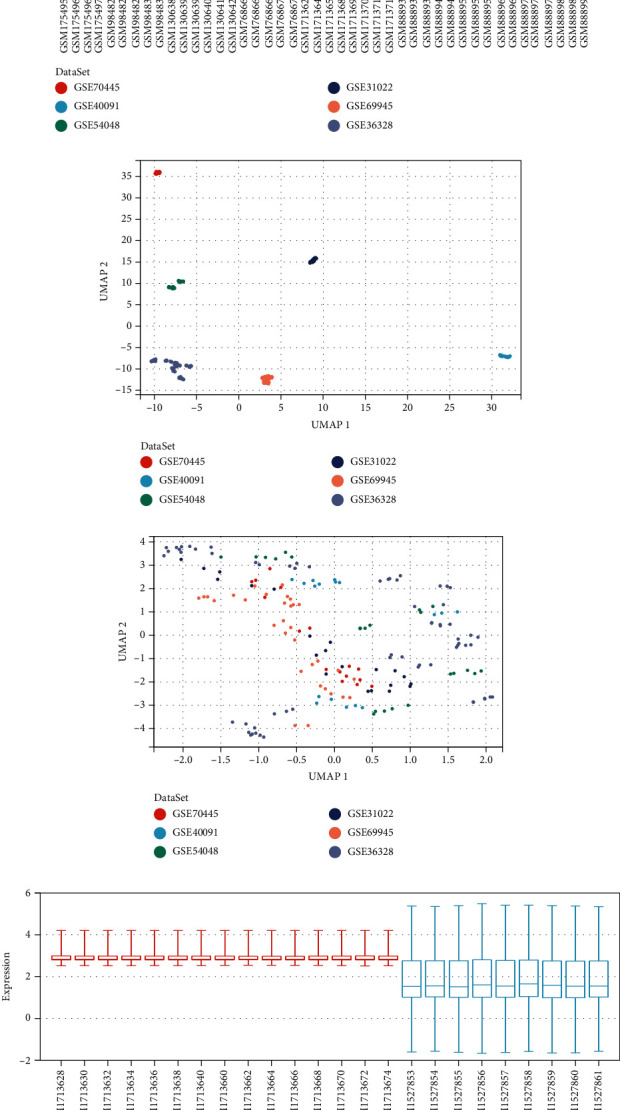
Dataset merging and moving batch effects. (a) UpSet plot of data intersection. (b, c) Boxplots of mRNA datasets before and after moving batch effect, sample number (*x*-axis) vs. gene expression (*y*-axis). (d, e) Umap of mRNA datasets before and after moving batch effect, UMAP1 (*x*-axis) vs. UMAP2 (*y*-axis). (f, g) Boxplots of miRNA datasets before and after moving batch effect, sample number (*x*-axis) vs. gene expression (*y*-axis). (h, i) Umap of miRNA datasets before and after moving batch effect, UMAP1 (*x*-axis) vs. UMAP2 (*y*-axis).

For the 6 mRNA datasets, we merged the matrices (Figure 1(a)), and as described above, we obtained merged dataset without batch effects. The data distribution among the various databases tends to be consistent, and the median was on a line (Figures 1(b) and 1(c)). And all sample points were evenly distributed together, suggesting a better removal of batch effects (Figures 1(d) and 1(e)).

Based on the same method, we also obtained the final data of miRNA (Figures 1(f)–1(i)).

### 3.2. Difference Analysis of DEGs and miRNA

**Figure 2:**
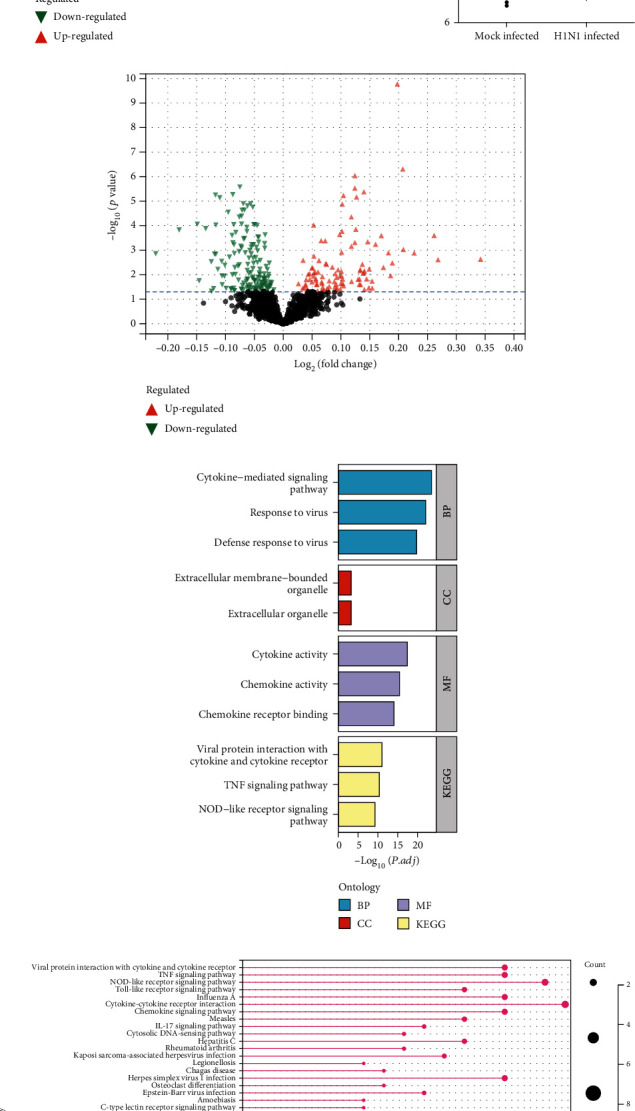
Gene Ontology and KEGG analysis results of differentially expressed genes associated with H1N1 infection. (a) Volcano plot of DEGs. (b) Boxplot of Cd274 expression difference between H1N1-infected group and mock-infected group. (c) Histogram of GO analysis for DEGs. (d) Volcano plot of DEmiRs. (e) Lollipop illustration of KEGG enrichment analysis for DEGs.

We analyzed microarray data using the limma package to screen for DEGs and miRNAs (DEmiRs) (Figures 2(a) and 2(c)). Finally, we obtained 114 DEGs including 100 upregulated and 14 downregulated genes. Also, we obtained 37 DEmiRs including 26 upregulated and 11 downregulated miRNAs (Table 2).

Following that, 114 DEGs were uploaded to the web-based tool DAVID to identify overrepresented GO categories and KEGG pathways. GO analysis results showed that DEGs were significantly enriched in multiple biological processes (BP), including response to the virus, defense response to the virus, and cytokine-mediated signaling pathway. DEGs were also involved in multiple molecular functions (MF), including cytokine activity, chemokine activity, and chemokine receptor binding. For cellular components, DEGs were involved in symbiont-containing vacuole membrane, symbiont-containing vacuole, and extracellular membrane-bounded organelle (Figure 2(b)). According to KEGG analysis, DEGs were enriched in viral protein interaction with cytokine and cytokine receptor, TNF signaling pathway, and NOD-like receptor signaling pathway (Figure 2(d)). Surprisingly, we found that DEGs were also enriched in PD-L1 expression and PD-1 checkpoint pathway (Figure 2(e)). In particular, Cd274 (PD-L1) was highly expressed in the H1N1-infected group (Figure 2(b)).

### 3.3. WGCNA Analysis

**Figure 3:**
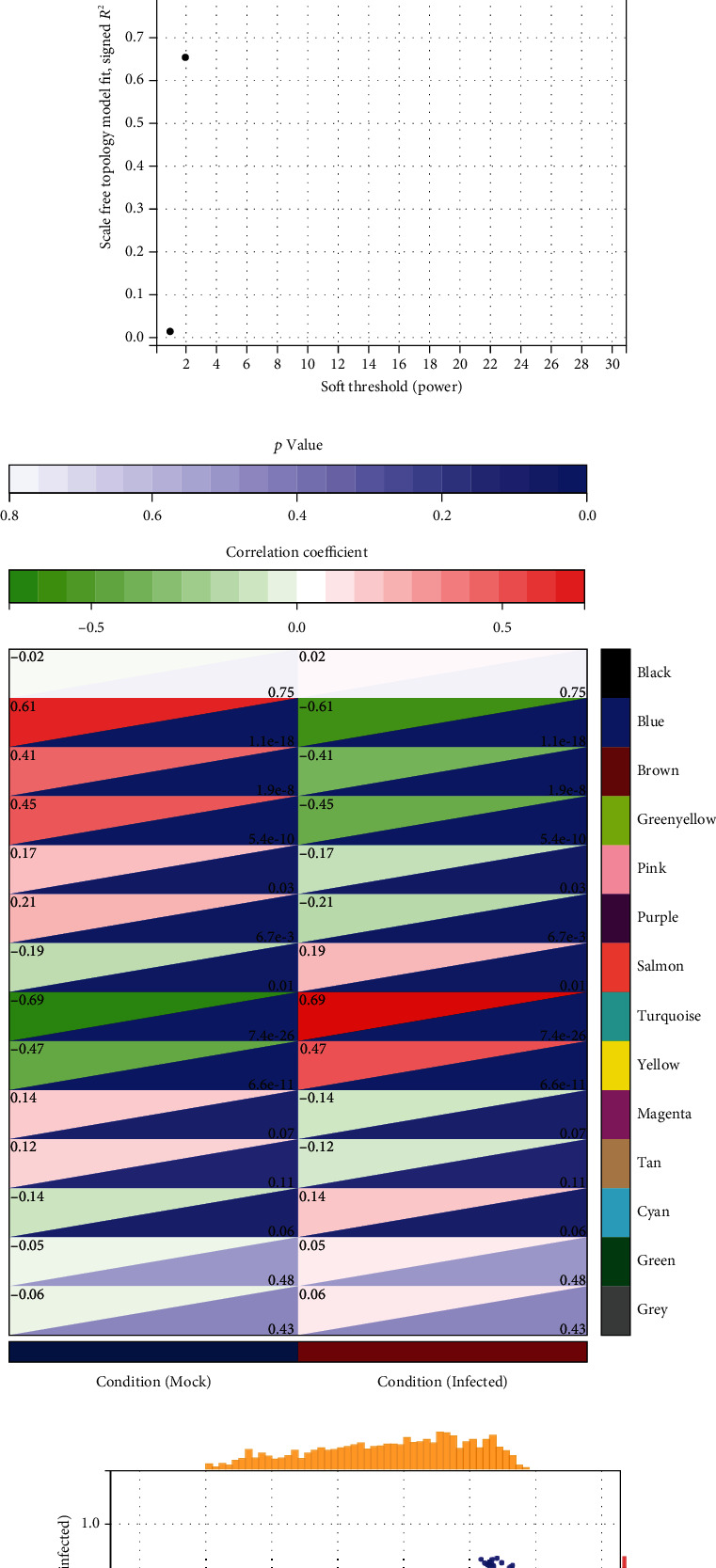
WGCNA analysis for mRNA datasets. (a) Dendrogram obtained by hierarchical clustering of genes based on their topological overlap. (b) The best soft threshold power for WGCNA analysis. (c) Each colored row represents a color-coded module that contains a group of highly connected genes. (d) Scatterplot shows a highly significant correlation between GS and MM with H1N1 infection in turquoise module.

All 174 samples and 7387 genes retrieved from the mRNA datasets were used for the coexpression network analysis. An eigengene correlation coefficient square and a soft threshold power of 4 were set to identify gene modules (Figure 3(b)). To further analyze the module, we calculated the dissimilarity of module eigengenes, chose a cut line for module dendrogram, and merged some module. Fourteen modules were identified when the DissThres was set as 0.25 after merging dynamic modules, as shown in the clustering dendrograms (Figure 3(a)). As shown in Figure 3(c), the turquoise module was significantly associated with H1N1 virus infection (correlation coefficient = 0.69). Based the cutoff criteria (|MM| > 0.8 and |GS| > 0.1), 286 genes with high connectivity in the turquoise module were identified as potential hub genes (Figure 3(d)).

**Figure 4:**
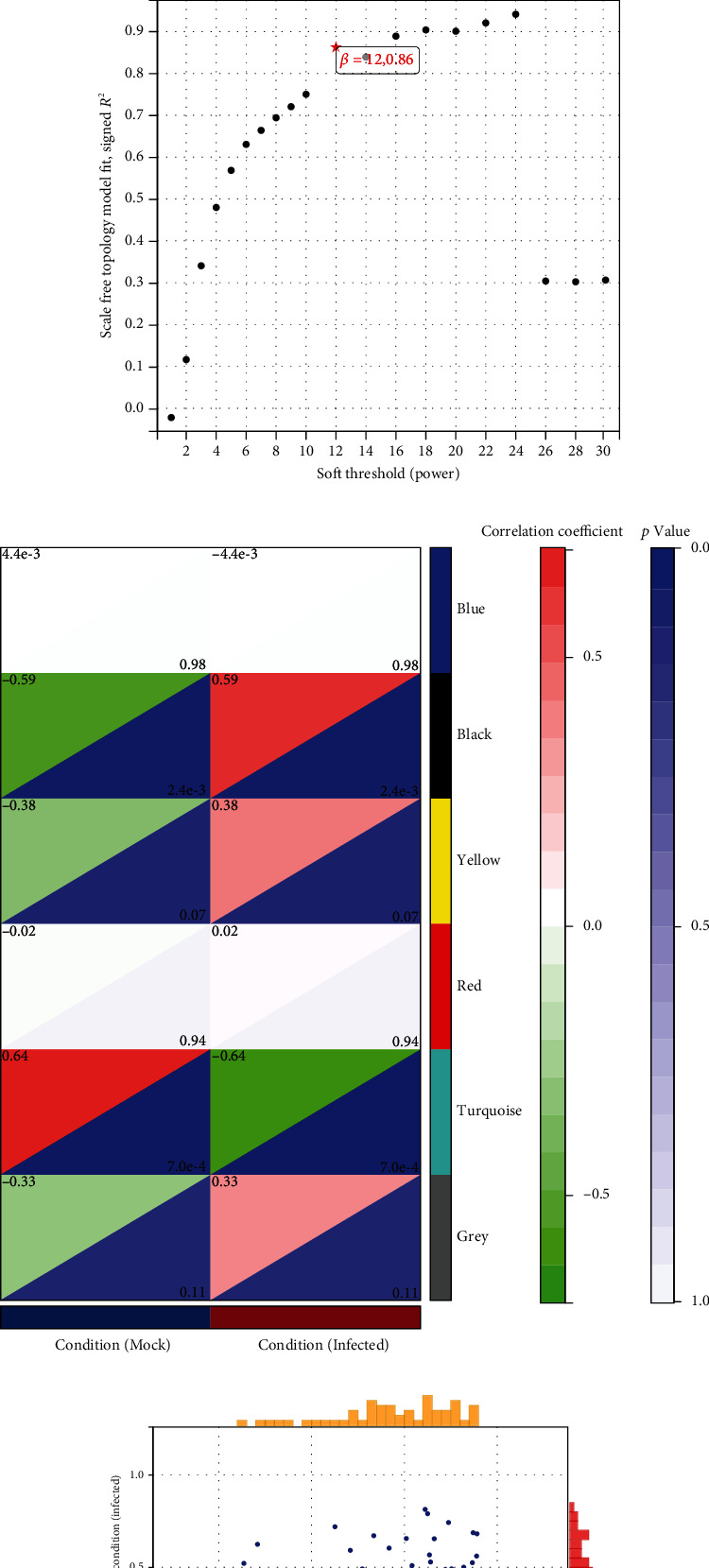
WGCNA analysis for miRNA datasets. (a) Dendrogram obtained by hierarchical clustering of genes based on their topological overlap. (b) The best soft threshold power for WGCNA analysis. (c) Each colored row represents a color-coded module that contains a group of highly connected genes. (d) Scatterplot shows a highly significant correlation between GS and MM with H1N1 infection in the black module.

Using the same method to analyze the miRNA dataset, 15 infected samples and 9 mock samples were included in the analysis (Figure 4(a)). With a soft threshold power of 12, we finally obtained 6 modules (Figure 4(b)). The black module was significantly associated with H1N1 virus infection (correlation coefficient = 0.59) (Figure 4(c)). In the black module, 27 miRNAs with high connectivity were identified as potential hub miRNAs (Figure 4(d)).

### 3.4. miRNA-mRNA Regulatory Networks and Hub Gene Analysis

**Figure 5:**
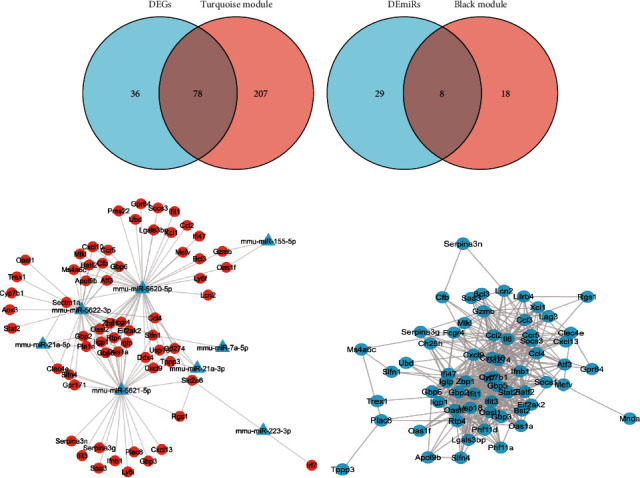
Regulatory networks in mice infected with H1N1 influenza virus. (a) Intersection of DEGs and turquoise module potential hub mRNAs. (b) Intersection of DEmiRs and black module potential hub miRNAs. (c) miRNA-mRNA regulatory network. (d) Protein-protein interaction network.

To further evaluate the interaction of DEGs and DEmiRs in mice after H1N1 influenza virus infection, we intersected turquoise module potential hub mRNAs with DEGs and black module potential hub miRNAs with DEmiRs (Figures 5(a) and 5(b)). A secondary list is obtained, which contains 78 candidate DEGs and 8 candidate DEmiRs. Further, we predicted the targets of 8 candidate DEmiRs by using the miRWalk database. After the intersection of these target genes and 78 candidate DEGs, 8 candidate DEmiRs targeted a total of 63 candidate DEGs (Table 3), and miRNA-mRNA regulatory network was constructed and output by Cytoscape software (Figure 5(c)).

On the other hand, we analyzed the 78 candidate DEGs according to the STRING database (Figure 5(d)). CytoHubba output results show three top real hub genes, which were Ifit3 (interferon-induced protein with tetratricopeptide repeats 2), Stat2 (signal transducer and activator of transcription 2), and Irf7 (interferon regulatory factor 7). These hub genes have a variety of biological activities such as RNA-binding activity, DNA-binding transcription factor activity, and RNA polymerase II-specific and *cis*-regulatory region sequence-specific DNA-binding activity (Table 4).

### 3.5. Validation of Four Real Hub Genes Expression Using High-Throughput Sequencing Data

**Figure 6:**
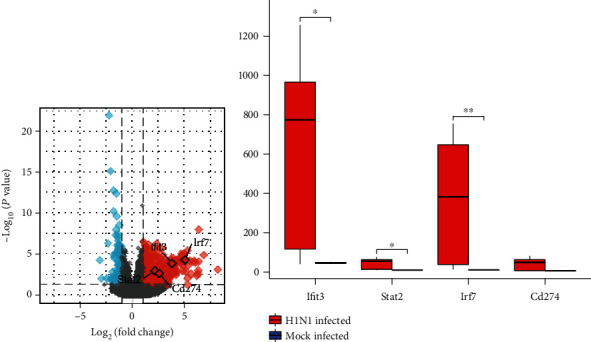
edgeR analysis of GSE98527 to validate real hub genes. (a) Volcano plot; real hub genes were highlighted. (b) Boxplot of real hub genes.

To validate the previous analysis results, we used another high-throughput sequencing dataset, GSE98527. We used edgeR package to analyze this dataset. The volcano plot showed that in GSE98527, Ifit3, Stat2, Irf7, and Cd274 were all high expression patterns (Figure 6(a)). But the box diagram showed that there was no significant difference in Cd274 expression between the two groups (Figure 6(b)).

## 4. Discussion

The new type A H1N1 virus discovered in 2009 has caused a worldwide epidemic and is transmitted from person to person through the respiratory tract by direct or indirect contact. The strain contains the genes of swine flu, avian flu, and human flu fragments [21]. In 1918, the Spanish influenza A (H1N1) influenza virus infected one-third of the world's population and had a mortality rate 25 times that of other influenza viruses. Worldwide, approximately 40 million people died from the virus [22]. To evaluate the impact of the H1N1 influenza virus on the host, research teams performed gene chip and high-throughput sequencing analysis on H1N1 influenza virus-infected mice [11–16]. In this study, we analyzed six mRNA microarray datasets and two miRNA datasets. In order to ensure the correctness of the analysis results, all datasets were normalized and have been moving batch effects. Additionally, we validated results using the high-throughput sequencing dataset GSE98527. Finally, we screened out 114 DEGs and 8 candidate DEmiRs.

Paquette et al. (contributors to GSE31022) found that infection of mice with A/Mexico/4108/2009 (H1N1pdm) resulted in elevated levels of IL-6 and was mediated through JAK/STAT3 signaling inflammatory response. DEGs were significantly enriched in three prominent functional clusters: cell growth and metabolism, interferon response, and inflammatory response [11]. Our combined analysis of five other datasets showed that DEGs were also significantly enriched in response to the virus, defense response to the virus, and negative regulation of JAK-STAT cascade and other multiple BP, which is also consistent with the results observed by Paquette et al. These DEGs were also involved in multiple molecular functions (MF), including cytokine activity, chemokine activity, and chemokine receptor binding. For cellular components, DEGs were involved in symbiont-containing vacuole membrane, symbiont-containing vacuole, and extracellular membrane-bounded organelle. More importantly, we found that DEGs were enriched in TNF signaling pathways, suggesting that TNF is an important indicator of H1N1 infection. Multiple meta-analyses have also revealed that TNF gene polymorphisms are associated with H1N1 virus susceptibility and severity of infection [22–26]. On the other hand, no elevated levels of TNF-related factors were detected in the plasma of pregnant women vaccinated against the H1N1 virus. However, the vaccine's components also contained HA, suggesting that vaccination against H1N1 virus is safe and effective against H1N1 virus infection [27].

Also, we found that DEGs were also enriched in PD-L1 expression and PD-1 checkpoint pathway. This was an unexpected discovery: Cd274 (PD-L1) was highly expressed in the H1N1-infected group (both in the training dataset and the validation dataset). PD-L1 is the ligand of PD-1 (programmed cell death 1). Various tumor cells evade antitumor immunity by overexpressing PD-L1 and utilizing PD-L1/PD-1 signaling [28]. Since PD-L1 upregulation inhibits the immune response of T cells to HBV, highly expressed PD-L1 is a negative regulator of the antiviral immune response [29]. At present, SARS-CoV-2 virus is spreading worldwide. The increased level of interferon-*γ* in the peripheral blood of patients further induces the enhanced expression of PD-L1 on the surface of T cells, making the immune checkpoint lose its inhibitory effect on the virus [30]. H1N1 virus can also cause acute respiratory infectious diseases, but no study has reported the relationship between highly expressed PD-L1 and H1N1 influenza virus infection.

Next, we screened three hub genes from the PPI network: Ifit3 (interferon-induced protein with tetratricopeptide repeats 3), Stat2 (signal transducer and activator of transcription 2), and Irf7 (interferon regulatory factor 7). Studies by Tran et al. have demonstrated that Ifit2, although an interferon-stimulated gene with well-established antiviral activity, can be exploited by the influenza virus to promote the translation of viral mRNA. IFIT1, IFIT2, and IFIT3 form hetero-oligomers and modulate each other's activities. Loss of IFIT3 also reduced influenza virus replication [31–33]. Our analysis found that Stat2 exhibited a high expression pattern after H1N1 infection in mice, which may be the transcriptional activation of related genes initiated by cellular pattern receptors in response to virus infection [34]. However, Jia et al. found that the influenza virus disrupts interferon signaling through its own nonstructural protein 1 (NS1) and inhibits nuclear translocation of phosphorylated STAT2 to enhance viral replication [35]. On the other hand, Irf7 plays an important role in the resistance to influenza A virus infection. If the Irf7 gene is deleted, it will greatly increase the susceptibility of the host to the H1N1 virus [36].

MicroRNAs (miRNAs) are important regulators of gene expression and usually play a role in degrading target genes by binding to 3′UTR of target genes [37]. However, gene expression regulation is an extremely complex network, and increasing evidence shows that miRNAs can also promote the expression of target genes [38]. In our analysis results, 8 candidate DEmiRs target 63 candidate DEGs. This indicated that H1N1 virus infection is a complex gene regulation process.

Therefore, this study used bioinformatics methods to analyze the changes in mRNA and miRNA in mice after H1N1 infection, suggesting that H1N1 influenza virus may promote the expression of CD274. Simultaneously, a miRNA-mRNA regulatory network was constructed. All analysis results will help researchers gain insights into the intrinsic effects of H1N1 influenza virus infection on the host and suggest a novel association of H1N1 virus with the host immune system.

## Figures and Tables

**Table 1:** Main information of the samples.

GEO DataSets ID	mRNA/miRNA	Experiment type platforms	H1N1 virus strain	Number of samples
GSE31022	mRNA	GPL6887	A/Mexico/4108/2009	18
GSE36328	mRNA	GPL7202	A/California/04/2009	9
			MA1-A/California/04/2009	9
			A/Mexico/4482/09	9
			A/Brisbane/59/07	7
			A/New Jersey/8/76	7
			1918 pandemic H1N1 influenza virus	9
GSE40091	mRNA		A/California/04/2009	9
GSE54048	mRNA		A/Mexico/4482/2009	15
GSE70445	mRNA		1918 pandemic H1N1 influenza virus	12
GSE69945	mRNA	GPL11202	A/California/04/2009	20
GSE69944	miRNA	GPL19970	A/California/04/2009	9
GSE62495	miRNA	GPL19117	A/Puerto Rico/8/34	6

**Table 2:** Differentially expressed gene and miRNA list.

Type	Condition	Number	Gene symbol (top 5)
DEGs	Upregulated	100	Saa3, Ccl4, Cxcl10, Cxcl1, and Ccl2
	Downregulated	14	Glb1l2, Aox3, Tff2, Neurog1, and Scgb3a1
DEmiRs	Upregulated	26	mmu-miR-21a-3p, mmu-miR-7a-5p, mmu-miR-223-5p, mmu-miR-5620-5p, and mmu-miR-147-3p
	Downregulated	11	mmu-miR-205-5p, mmu-miR-31-3p, mmu-miR-3060-3p, mmu-miR-224-3p, and mmu-miR-744-3p

**Table 3:** Targets of candidate DEmiRs.

miRNA	Target genes
mmu-miR-21a-3p	Ccl4, Rgs1, Cd274, and Tppp3
mmu-miR-7a-5p	Slfn1
mmu-miR-5620-5p	Atf3, Socs3, Cfb, Ifi47, Ifit1, Xcl1, Ly6f, Cxcl9, Ccr5, Ddx4, Gbp2, Lgals3bp, Eif2ak2, Ccl2, Slfn1, Oasl2, Usp18, Gzmb, Ccl4, Igtp, Mefv, Cxcl10, Cd274, Ubd, Ms4a6c, Batf2, Mlkl, Gpr84, Bcl3, Zbp1, Apol9b, Slc2a6, Prss22, Pla1a, Fcgr4, Oas1f, Oas1a, Sectm1a, Gbp5, Gbp6, Rtp4, Iigp1, Tppp3, and Lcn2
mmu-miR-223-3p	Slc2a6, Irf7
mmu-miR-5621-5p	Cxcl9, Serpina3n, Ifnb1, Rgs1, Igtp, Cxcl13, Oas1a, Tppp3, Eif2ak2, Slfn4, Usp18, Ly6i, Serpina3g, Pla1a, Gpr171, Plac8, Gbp2, Ifit3, Saa3, Slfn1, Gbp3, Clec4e, Zbp1, Cd274, Rtp4, Ddx4, Slc2a6, Fcgr4, Gbp5, Iigp1, and Oasl2
mmu-miR-155-5p	Oas1f, Gzmb
mmu-miR-5622-3p	Gbp2, Eif2ak2, Slfn1, Clec4e, Stat2, Cxcl10, Zbp1, Ms4a6c, Cfb, Iigp1, Apol9b, Fcgr4, Oas1a, Sectm1a, Gpr171, Gbp6, Slfn4, Igtp, Oasl1, Atf3, Cyp7b1, Ccr5, Trex1, Oasl2, Ccl4, Aox3, Batf2, Mlkl, Pla1a, Gbp5, and Rtp4
mmu-miR-21a-5p	Clec4e, Sectm1a, Pla1a, and Gbp2

**Table 4:** Main information of real hub genes.

Rank	Gene symbol	Full name	Function
1	Ifit3	Interferon-induced protein with tetratricopeptide repeats 3	Predicted to enable RNA-binding activity and to be involved in interferon-beta pathway.
2	Stat2	Signal transducer and activator of transcription 2	Involved in negative regulation of type I interferon-mediated signaling pathway and type I interferon signaling pathway.
3	Irf7	Interferon regulatory factor 7	Enables DNA-binding transcription factor activity, RNA polymerase II-specific and *cis*-regulatory region sequence-specific DNA-binding activity.

## Data Availability

The data used to support the findings of this study are included within the article.

## References

[1] Rossman J. S., Lamb R. A. (2011). Influenza virus assembly and budding. *Virology*.

[2] Shao W., Li X., Goraya M. U., Wang S., Chen J. L. (2017). Evolution of influenza A virus by mutation and re-assortment. *International Journal of Molecular Sciences*.

[3] Treanor J. (2004). Influenza vaccine-outmaneuvering antigenic shift and drift. *The New England Journal of Medicine*.

[4] Fiers W., De Filette M., Birkett A., Neirynck S., Min J. W. (2004). A "universal" human influenza A vaccine. *Virus Research*.

[5] Hagau N., Slavcovici A., Gonganau D. N. (2010). Clinical aspects and cytokine response in severe H1N1 influenza A virus infection. *Critical Care*.

[6] de Jong M. D., Simmons C. P., Thanh T. T. (2006). Fatal outcome of human influenza A (H5N1) is associated with high viral load and hypercytokinemia. *Nature Medicine*.

[7] Iwasaki A., Pillai P. S. (2014). Innate immunity to influenza virus infection. *Nature Reviews Immunology*.

[8] Yip T. F., Selim A. S. M., Lian I., Lee S. M. (2018). Advancements in host-based interventions for influenza treatment. *Frontiers in Immunology*.

[9] Acuña S. M., Floeter-Winter L. M., Muxel S. M. (2020). MicroRNAs: biological regulators in pathogen-host interactions. *Cell*.

[10] Yasui M., Kunita A., Numakura S., Uozaki H., Ushiku T., Fukayama M. (2020). Cancer stem cells in Epstein-Barr virus-associated gastric carcinoma. *Cancer Science*.

[11] Paquette S. G., Banner D., Zhao Z. (2012). Interleukin-6 is a potential biomarker for severe pandemic H1N1 influenza A infection. *PLoS One*.

[12] Josset L., Belser J. A., Pantin-Jackwood M. J. (2012). Implication of inflammatory macrophages, nuclear receptors, and interferon regulatory factors in increased virulence of pandemic 2009 H1N1 influenza A virus after host adaptation. *Journal of Virology*.

[13] Morrison J., Josset L., Tchitchek N. (2014). H7N9 and other pathogenic avian influenza viruses elicit a three-pronged transcriptomic signature that is reminiscent of 1918 influenza virus and is associated with lethal outcome in mice. *Journal of Virology*.

[14] Go J. T., Belisle S. E., Tchitchek N. (2012). 2009 pandemic H1N1 influenza virus elicits similar clinical course but differential host transcriptional response in mouse, macaque, and swine infection models. *BMC Genomics*.

[15] Feng S., Heath E., Jefferson B. (2021). Hypergraph models of biological networks to identify genes critical to pathogenic viral response. *BMC Bioinformatics*.

[16] Walters K. A., D'Agnillo F., Sheng Z. M. (2016). 1918 pandemic influenza virus and Streptococcus pneumoniae co-infection results in activation of coagulation and widespread pulmonary thrombosis in mice and humans. *The Journal of Pathology*.

[17] Johnson W. E., Li C., Rabinovic A. (2007). Adjusting batch effects in microarray expression data using empirical Bayes methods. *Biostatistics*.

[18] Langfelder P., Horvath S. (2008). WGCNA: an R package for weighted correlation network analysis. *BMC Bioinformatics*.

[19] Tang J., Kong D., Cui Q. (2018). Prognostic genes of breast cancer identified by gene co-expression network Analysis. *Oncologia*.

[20] Sticht C., De La Torre C., Parveen A., Gretz N. (2018). miRWalk: An online resource for prediction of microRNA binding sites. *PLoS One*.

[21] Yang N., Hong X., Yang P. (2011). The 2009 pandemic A/Wenshan/01/2009 H1N1 induces apoptotic cell death in human airway epithelial cells. *Journal of Molecular Cell Biology*.

[22] Horimoto T., Kawaoka Y. (2005). Influenza: lessons from past pandemics, warnings from current incidents. *Nature Reviews Microbiology*.

[23] García-Ramírez R. A., Ramírez-Venegas A., Quintana-Carrillo R., Camarena Á. E., Falfán-Valencia R., Mejía-Aranguré J. M. (2015). TNF, IL6, and IL1B polymorphisms are associated with severe influenza A (H1N1) virus infection in the Mexican population. *PLoS One*.

[24] Li Y., Chen X. Y., Gu W. M. (2020). A meta-analysis of tumor necrosis factor (TNF) gene polymorphism and susceptibility to influenza A (H1N1). *Computational Biology and Chemistry*.

[25] Elsayed S. M., Hassanein O. M., Hassan N. H. A. (2019). Influenza A (H1N1) virus infection and TNF-308, IL6, and IL8 polymorphisms in Egyptian population: a case-control study. *The Journal of Basic and Applied Zoology*.

[26] Alagarasu K., Kaushal H., Shinde P. (2021). TNFA and IL10 polymorphisms and IL-6 and IL-10 levels influence disease severity in influenza A(H1N1)pdm09 virus infected patients. *Genes*.

[27] Dias F. M. V., Diniz M. F., Franco G. C. (2018). Effects of vaccination against the H1N1 virus on BDNF and TNF-*α* plasma levels in pregnant women. *Current Drug Safety*.

[28] Cha J. H., Chan L. C., Li C. W., Hsu J. L., Hung M. C. (2019). Mechanisms controlling PD-L1 expression in cancer. *Molecular Cell*.

[29] Liu L., Hou J., Xu Y. (2020). PD-L1 upregulation by IFN-*α*/*γ*-mediated Stat1 suppresses anti-HBV T cell response. *PLoS One*.

[30] Aghbash P. S., Eslami N., Shamekh A., Entezari-Maleki T., Baghi H. B. (2021). SARS-CoV-2 infection: the role of PD-1/PD-L1 and CTLA-4 axis. *Life Sciences*.

[31] Pichlmair A., Lassnig C., Eberle C. A. (2011). IFIT1 is an antiviral protein that recognizes 5′-triphosphate RNA. *Nature Immunology*.

[32] Fleith R. C., Mears H. V., Leong X. Y. (2018). IFIT3 and IFIT2/3 promote IFIT1-mediated translation inhibition by enhancing binding to non-self RNA. *Nucleic Acids Research*.

[33] Johnson B., VanBlargan L. A., Xu W. (2018). Human IFIT3 modulates IFIT1 RNA binding specificity and protein stability. *Immunity*.

[34] Saito T., Gale M. (2007). Principles of intracellular viral recognition. *Current Opinion in Immunology*.

[35] Jia D., Rahbar R., Chan R. W. (2010). Influenza virus non-structural protein 1 (NS1) disrupts interferon signaling. *PLoS One*.

[36] Wilk E., Pandey A. K., Leist S. R. (2015). RNAseq expression analysis of resistant and susceptible mice after influenza A virus infection identifies novel genes associated with virus replication and important for host resistance to infection. *BMC Genomics*.

[37] Stavast C. J., Erkeland S. J. (2019). The non-canonical aspects of microRNAs: many roads to gene regulation. *Cell*.

[38] Pu M., Chen J., Tao Z. (2019). Regulatory network of miRNA on its target: coordination between transcriptional and post-transcriptional regulation of gene expression. *Cellular and Molecular Life Sciences*.

